# Building Environmental Health and Genomics Literacy among Healthcare Providers Serving Vulnerable Communities: An Innovative Educational Framework

**DOI:** 10.3390/ijerph19020929

**Published:** 2022-01-14

**Authors:** Kathleen Mead Vandiver, Esther Erdei, Amanda G. Mayer, Catherine Ricciardi, Marcia O’Leary, Kathleen Burke, Judith T. Zelikoff

**Affiliations:** 1 MIT Center for Environmental Health Sciences, MIT Superfund Research Program, and the MIT Edgerton Center, Massachusetts Institute of Technology, Cambridge, MA 02139, USA; 2 University of New Mexico Health Sciences Center, College of Pharmacy, Albuquerque, NM 87106, USA; EErdei@salud.unm.edu; 3 MIT Edgerton Center, Massachusetts Institute of Technology, Cambridge, MA 02139, USA; angruhl@mit.edu; 4 MIT Center for Clinical and Translational Research, Massachusetts Institute of Technology, Cambridge, MA 02139, USA; c_ricci@mit.edu; 5 Missouri Breaks Industries Research Inc., Eagle Butte, SD 57625, USA; marcia.oleary@mbiri.com; 6 Ramapo College Nursing, Ramapo College of New Jersey, Mahwah, NJ 07430, USA; kmburke@ramapo.edu; 7 Department of Environmental Medicine, New York University Grossman School of Medicine, New York, NY 10016, USA

**Keywords:** environmental exposures, nursing education, genomics, hands-on learning, community health, genetic susceptibility, Indigenous populations

## Abstract

This study addresses healthcare providers’ knowledge deficits in environmental health and genetics, and primarily focuses on student nurses and nurses serving marginalized, low-income communities frequently exposed to environmental toxicants. Our approach to improve public health is unique, combining hands-on modeling exercises with case-based lessons in addition to three targeted 40 min lectures on toxicology. These lectures included the team’s community-based environmental health research among Indigenous peoples of the U.S. The hands-on approach employed DNA and protein molecular models designed to demonstrate normal and dysfunctional molecules, as well as genetic variants in world populations. The models provided learners with visuals and an experience of “learning by doing.” Increased awareness of the effects of environmental toxicants is the first step toward improving health care for exposed communities. We measured knowledge gains by pre- and post-tests among student nurses and nurses serving Native Americans living both in urban and rural areas of the U.S. (*n* = 116). The modeling lessons illustrated genetic variants in liver proteins common in Native peoples and their resulting health vulnerabilities. Participants were engaged and enthusiastic; and pre- and post-test results reported substantial knowledge gains and a greater understanding of genetic susceptibility (*p* < 0.0001). Our study demonstrates the utility of this framework across diverse populations and remote communities.

## 1. Introduction

Environmental exposures profoundly affect human health, but community health professionals, such as physicians, nurses, and nursing assistants, lack basic training in environmental health. Compounding this, there is a lack of awareness of genetic susceptibility and its interaction with environmental health hazards [1,2,3]. Such susceptibilities can be specific to different world populations and particular ethnicities. A lack of knowledge is evident among clinical providers serving Tribal community members in the United States. This is especially true in the Western and Midwestern states where more than 600,000 Native community members are exposed to mountain-top mining waste [4]. Moreover, other Tribal communities have their Tribal lands in close proximity to heavily contaminated U.S. EPA-designated waste sites. Indeed, 13% of the 1344 Superfund sites in the U.S. have a reservation within 10 miles and as of 2013, about 7.5% of Native American homes did not have safe drinking water or basic sanitation [5,6,7,8]. These same communities also struggle with many gene-associated, environmentally-induced chronic diseases, such as high rates of type II diabetes, cardiovascular diseases, stroke, and several types of cancers [9,10,11,12,13,14]. Therefore, education of health professionals is essential as the first step in improving community health with the ultimate goal of translating this knowledge to the entire community. Thus, a multidisciplinary collaboration among three academic research centers joined with nursing professionals working with Tribal nations for the purpose of piloting a new educational approach to meet community needs. This environmental justice work is significant because it fills a much-needed gap in health delivery and disease prevention. By elucidating complex genetic interactions in health and extending the reach of these understandings, preventive strategies are more likely to be implemented to improve public health for marginalized communities, including indigenous peoples throughout the world.

The National Institutes of Health (NIH) distinguishes between the terms of genetics and genomics in the following way. Genetics is “the study of all of a person’s genes and their inheritance of disease.” Genomics, however, involves “the study of all of a person’s genes including the study of the interaction of genes with each other and with the environment” [15]. Since genomics is inclusive of gene-environment interactions, this term will be used hereafter in this context. The Precision Medicine Initiative is a future-looking goal in the U.S. that seeks to perfect prevention and treatment strategies by taking individual genomic variability into account. This movement emphasizes the relevance of genomics in nurses’ clinical practice, and points out that nurses need more training and greater confidence in genomics to fully participate in this effort. The rapid pace of scientific discoveries makes educational progress difficult, but nursing education needs to evolve and integrate genomics into the regulatory standards [16]. The U.S. is not alone in documenting the need for integration of genomics teaching into nursing education, as this is a worldwide issue, including in Hong Kong, Taiwan, and Mainland China [17]. One major milestone that has been met in the U.S. is the development of the required essential nursing educational competencies in genetics and genomics [18].

Our novel educational method introduced here includes short modules with modeling kits, providing hands-on learning experiences that faculty at nursing schools can insert into their existing coursework. In the future, these modules could be tailored for self-study and used by professional clinicians and nurses, as well as academic faculty members, to fill continuing educational needs. Here, because our focus was on nursing students’ learning gains after working with unique tactile molecular models, we reviewed learning style preferences of students in nursing schools. Dr. Santhamma James et al. [19], reporting on learning style preferences of their first-year nursing students, found that tactile learning was ranked the highest, above visual, aural, and read–write methods by students (*p* < 0.001). They also noted that rural students had significantly higher preference for visual and tactile learning compared to their metropolitan counterparts. Based on these and additional study results, the authors called for teaching in nursing schools to be both more hands-on and more interactive to improve the effectiveness of the learning process. The overall effects of learning style on the performance of university students have also been well documented by numerous researchers [20,21,22].

Kathleen Calzone and collaborators in 2018 acknowledged that a concerted global effort was needed to address well-known deficits in nurses’ genomic literacy. They defined three primary educational challenges: insufficient curriculum time, insufficient educators capable of teaching genomics (which includes both genetics and environmental health connections), and absence of the competency assessments required for nursing. Only one country (Israel) required a demonstration of genetic/genomic knowledge for general nursing practice [23]. A survey of 23 nurse leaders from 18 countries revealed that genomic knowledge was basically confined to specialists associated with newborn screening programs, and that worldwide, genomics was often viewed as a niche specialty [24]. In contrast, Calzone and colleagues in the International Society of Nurses in Genetics (ISONG) view genomics as relevant to all healthcare providers, as it “provides an underpinning for understanding health, risks for manifestations of disease, therapeutic decisions, development of new therapies, and responses to interventions” [23]. The World Health Organization also affirms nursing’s leadership role, stating that as the largest single healthcare professional group worldwide, nurses have a pivotal role in bringing the benefits of genomics to everyday healthcare [25].

Some countries have created high-quality, national programs in genomics to fill the gaps in the environmental health and genetics knowledge for the nursing profession. For example, the U.S. National Institute of Nursing Research’s Summer Genetics Institute (SGI) was established about 18 years ago, and annually provides intensive laboratory training in molecular genetics for a group of nurse researchers. However, to bring the benefits of genomics education to everyday health care, which is the goal, broader programs for greater numbers of nurses need to be accommodated [18].

The main aim of this study was to pilot a novel environmental health and genetics learning framework by combining an interactive, hands-on modeling activity with classical lecture experiences. The purpose was also to demonstrate effective communication of key concepts in environmental health and genetics (e.g., genomics) to health professionals, building the groundwork for translating these concepts to their community members in culturally appropriate language. Our hands-on approach employed the unique MIT DNA and protein models that were designed to provide the users with visual, tactile, and “learning by doing” experiences. With the provided models, participants could independently explore and verify key concepts, generating greater engagement and understanding. In conclusion, this interactive learning experience increased literacy in genomics, and provided insight into how toxic agents are metabolized and how the process is influenced by genetics to produce a wide range of health outcomes. Specifically, the hands-on approach improved environmental health literacy as measured by pre- and post-tests with the participants demonstrating significant learning gains (*p* < 0.0001). This new approach was successful in building faculty instructors’ and students’ confidence in explaining the molecular mechanisms underlying the actions of environmental chemicals in the body.

## 2. Materials and Methods

The materials employed in the hands-on modeling study were the MIT Edgerton Center DNA and Protein Sets. They are described here along with the instructional methods used with the nurse study participants. The pre- and post-test materials used to measure nurses’ learning gains with the hands-on educational materials are also included in this section. Since this framework for building environmental health literacy includes lectures on toxicology and exposure biology to accompany the modeling experience, these materials will be briefly described here as well. For nurse audiences, the lectures provided context to the hands-on methods session, focusing on environmental health and impacts on environmental justice communities. For community audiences that included, for example, classroom groups seated in a school auditorium for a lecture, a simple survey form was piloted to assess their learning gains and increased interest in the lecture topic.

### 2.1. Model Development and Availability

Vandiver invented a flexible double helix model capable of revealing the functional aspects of DNA. The double helix can be used to visualize structural relationships, or easily opened into separate strands and laid flat to simulate cell processes. This functional prototype was first designed from off-the-shelf components. The models used in this research study have now been mass-produced and hold MIT patents. The MIT DNA/RNA kits became available for non-profit purchase in 2016; the protein kits and tRNA kits followed in January 2017. All three modeling kits are currently available and sold online in classroom sets of 14 kits, with each kit typically serving two students. The DNA/RNA models are used in public school districts around the continental U.S. and have been introduced globally. The sets are already serving educational institutions in Singapore, China, Switzerland, Spain, Mexico, Hawaii, and India. MIT’s overall mission is improving science education worldwide and MIT’s non-profit status makes it possible for the MIT Edgerton Center to offer these molecular models at cost with additional support from the NIEHS P30 Environmental Health Research Excellence Centers.

In planning manufacture for global dissemination, the MIT DNA and Protein Sets are well positioned for such distribution, as the plastic components are already mass produced through injection-molded processes where production rates can readily be increased. Instructors in different countries have observed that students with limited English proficiency can follow the written information in the instructional booklets, as the sentences are short and accompanied by photos/diagrams. Additionally, the booklets use scientific vocabulary sparingly, so teachers can add the scientific language that their students are required to learn.

The MIT authors originally created both basic and advanced booklets for teaching how cells make proteins from DNA instructions. More recently, the authors created additional instructional materials that are shown here for teaching hands-on lessons in toxicology using the existing MIT Edgerton Center’s molecular kits. Specifically, new lessons in environmental health were created to demonstrate how genetics play a critical role in a populations’ susceptibility to environmental exposures. In this educational research study, the nursing students initially performed several introductory activities to become familiar with how the models work and to learn the basics about protein form and function. First, they constructed a simplified cell membrane protein using the amino acid subunits. Then, using additional hands-on components, the participants successfully produced the same membrane protein from a gene, using the multiple steps occurring in protein synthesis. After the preparatory steps, students were introduced to a genetic case study in exposure biology. Because the purpose of the study was to evaluate the new instructional capability provided by the functional 3D molecular models, the models are described below in greater detail.

### 2.2. DNA/RNA and Protein Subunits

**Figure 1:**
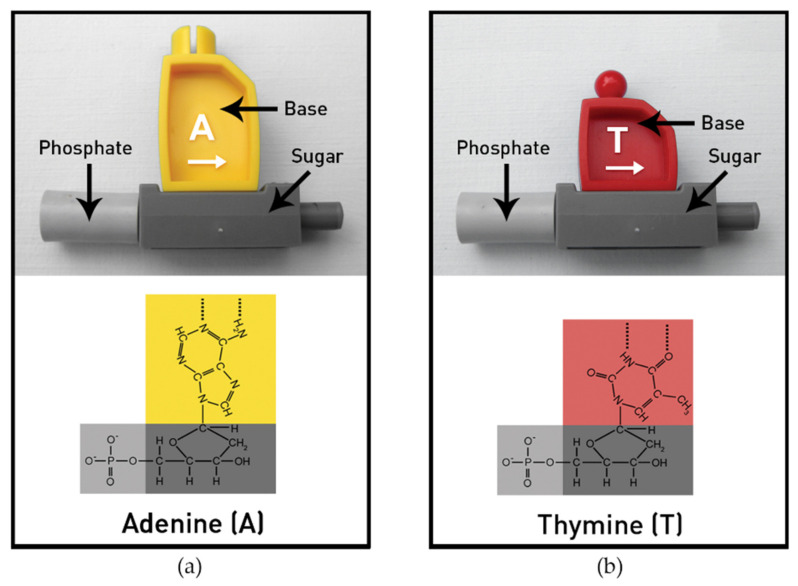
Diagram of the model nucleotides with chemical structures. (**a**) Model and chemical structure of the nucleotide adenine. Purines have larger bases, as shown in the model. (**b**) Model and chemical structure of the nucleotide thymine. Pyrimidines have smaller bases, as shown in the model. © MIT Edgerton Center DNA/RNA Booklet 1.

The pieces for the models are manufactured by an injection-molded process using ABS plastic with tight quality control and dimensional tolerance. The nucleotide monomers in the DNA/RNA kits can be linked and unlinked to demonstrate cell processes. The DNA nucleotide models are easily formed into long DNA strands by linking the phosphate and sugar components of adjoining nucleotides (Figure 1). Each base (thymine, adenine, cytosine, and guanine) has a designated color for ease of recognition, and the purine and pyrimidine bases are sized to resemble the differences in nature. The nucleotide sugars have a number 3′ (not visible on Figure 1) marked on the end that is opposite to the 5′ phosphate group. To further emphasize that two DNA strands should be constructed in opposite directions (antiparallel), the individual bases of both RNA and DNA are marked with prominent arrows pointing toward the 3′ end of the sugar. The large directional arrows in Figure 1 are marked in white. The 3′ biochemical designations inform advanced students, while novices correctly assemble an antiparallel double helix following the large arrows imprinted on the bases.

**Figure 2:**
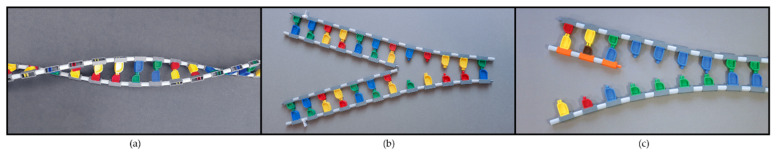
DNA and RNA nucleotides illustrate the utility of the models in teaching both structure and function. (**a**) The helical shape with the two antiparallel DNA strands. (**b**) Semiconservative DNA replication with a simplified replication fork. (**c**) The start of transcription with the insertion of the first three nucleotides. © MIT Edgerton Center DNA/RNA Booklet 1.

Base-pairing of the model nucleotides is accomplished through a ball-and-socket connection, permitting the two DNA strands to twist into a double helix as shown in Figure 2a. This same connection permits the two sides of the DNA ladder to easily open up when lying flat, demonstrating the weak hydrogen bonds that hold the two DNA strands together. A quick disconnect feature allows for the two DNA strands to open up easily along any section of the DNA ladder to demonstrate the cellular processes of replication, transcription, and DNA repair. A simplified DNA replication fork is shown in Figure 2b where two small white tags were added to each strand of original DNA. These tags allow participants to observe DNA replication and to discover the semiconservative nature of the process (one strand in the new molecule is always conserved from the original DNA). The process of transcription can also be accomplished using the RNA nucleotides provided in the DNA/RNA kit (Figure 2c). The strand opposite the gene serves as the template for the mRNA nucleotides. The resulting mRNA sequence matches the sequence of the gene.

Importantly, all the models work together. This includes the DNA, RNA, and tRNA pieces as well as the amino acids in the protein kit. Therefore, protein chains can be produced from mRNA transcripts and the models can also be used to demonstrate the process of translation on the ribosome. These modeling features provide learners with a seamless way to visualize numerous cell processes in molecular biology, and provide the designers with opportunities to publish additional instructional materials related to environmental health.

**Figure 3:**
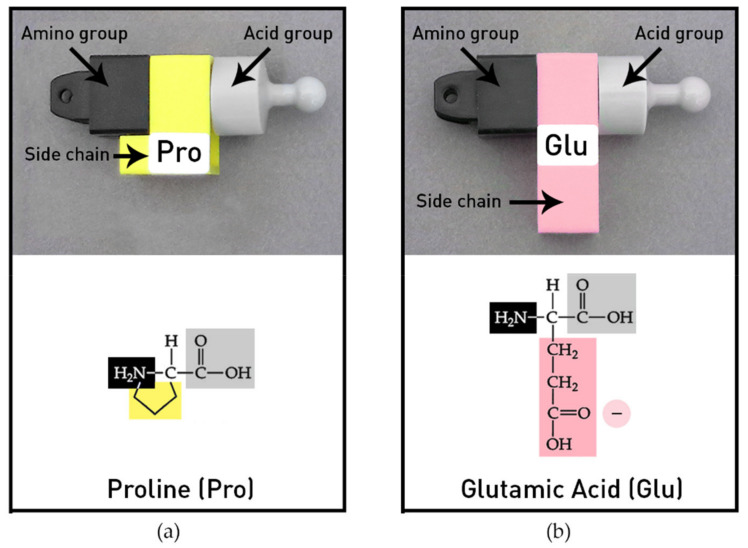
Two of the twenty different kinds of amino acids in the modeling kit are shown. (**a**) Proline is an atypical amino acid with an extra bond that connects back to the amino group. It is one of many hydrophobic (yellow) amino acids. (**b**) Glutamic acid, a typical hydrophilic amino acid with a long side pink chain. The colors of the side chain indicate the four chemical groups: hydrophobic amino acids are yellow (no charge), the hydrophilic amino acids are blue (positive charge), pink (negative charge), and green (polar with negative and positive charges). The chemical diagrams illustrate how the side chains (-R groups) were stylized in the models to indicate relative differences in size and shape. © MIT Edgerton Center Protein Booklet 1.

The amino acid models provided in the MIT Protein Set represent the most common twenty amino acids with many distinctive functional features. The individual amino acids have generalized side chains (-R groups) with shapes and colors to represent their different chemical properties. Amino acids can be joined end to end (amino group to acid group) by peptide bonds to create different sequences and chain lengths. The model protein chains are flexible and can be folded into classical biochemical forms, such as helices and beta pleated sheets, owing to the design of the amino acids’ side chains that can rotate freely, with the exception of proline. Proline’s side chain has an extra bond and thus its side chain cannot rotate (Figure 3a). Two of the twenty common amino acids in the protein kit are shown in Figure 3. The colors of the side chains indicate the four chemical groups. Hydrophobic amino acids are yellow and the hydrophilic amino acids include three colors: basic amino acid side chains are blue (positive), acid side chains are pink (negative), and polar side chains are green (polar). The chemical diagrams illustrate how the side chains (-R groups) were stylized to indicate relative differences in size and shape in the models.

### 2.3. Form and Function

**Figure 4:**
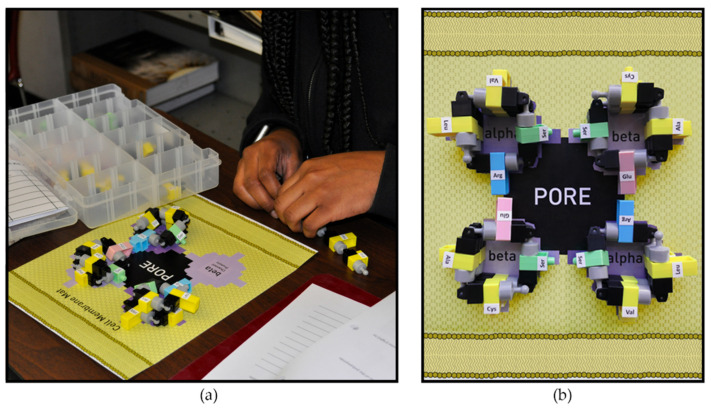
Channel protein model assembly on the cell membrane mat. (**a**) Participants assemble amino acids into a protein chain, following a specific sequence and fold the chain into an alpha helix with the side chains (-R groups) pointing toward the outside of the helices. (**b**) Top-down view of a cell membrane model with a functional channel protein represented by four amino acid chains in four helices spanning the cell membrane. This pore is functional because the hydrophilic amino acids face towards the interior of the pore, expediting the passage of ions through the cell membrane. The hydrophobic amino acids in the helices all face the hydrophobic lipids in the surrounding cell membrane. The protein’s function depends upon the order of the amino acids in the chain.

To learn how the amino acid sequence can affect function of the channel proteins in membranes, participants assembled individual amino acids into a protein chain with a prescribed sequence and then coiled each chain into a helix; in Figure 4, the side chains (-R groups) of the amino acids face outwards (towards their surroundings). For the next step, the participants decided how to position all four of these transmembrane proteins into the model cell membrane to create a working pore. A functioning channel protein will have hydrophilic amino acids facing inwards, to create the pore opening. These charged positive and negative side chains on the amino acids facilitate the passage of ions through the cell membrane. Figure 4b shows a top-down view of a cell membrane in which a channel protein is represented by four short amino acid chains spanning the thickness of lipid bilayer. In reality, a protein chain in a cell membrane has ten helical turns as opposed to two shown in this lesson. This simplified protein permits learners to later assemble the same channel protein starting from a gene and using the molecular processes of transcription and translation. The lesson plan also includes genes for demonstrating how a single nucleotide substitution can produce a non-functional membrane protein, as well as a second example where a single nucleotide substitution codes for the same amino acid and the protein remains functional.

### 2.4. Protein Synthesis

**Figure 5:**
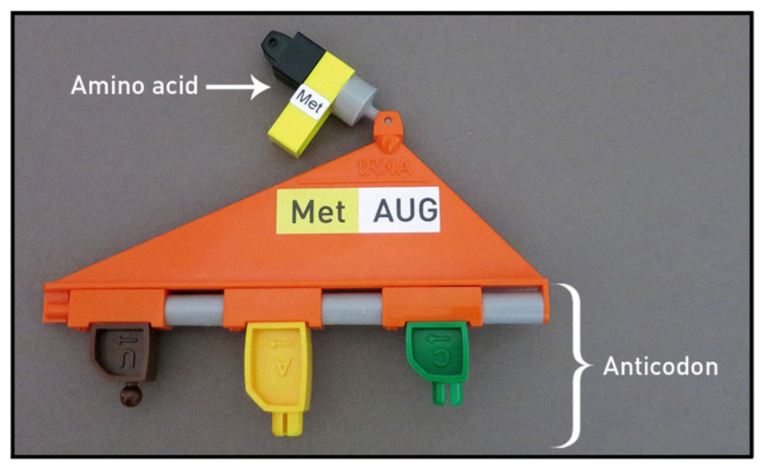
Example of a transfer RNA (tRNA) with amino acid attached. This tRNA molecule with its anticodon UAC carries only methionine (Met). © MIT Edgerton Center tRNA Booklet 1.

**Figure 6:**
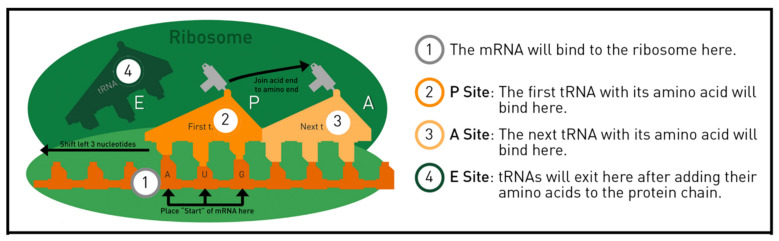
The ribosome mat shows the E, P, and A sites, as well as the mRNA’s position. With this mat, students can place the models into position and reenact the steps in protein synthesis. © MIT Edgerton Center tRNA Booklet 1.

**Figure 7:**
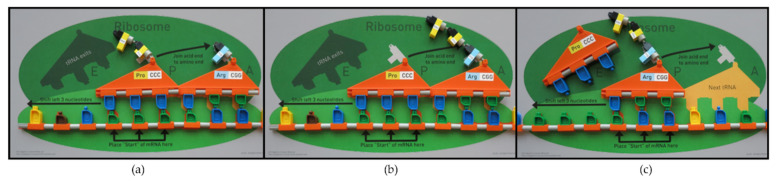
Peptide bonds form between the amino acids during translation on the ribosome, lengthening the protein chain. Refer to the descriptions in the text for explanations of (**a**–**c**). © MIT Edgerton Center tRNA Booklet 1.

The tRNA kit, working together with the other models and materials, demonstrates how messenger RNA (mRNA) determines the order of the amino acids creating a new protein on the ribosome. As shown in Figure 5, each tRNA carries a specific amino acid. The RNA nucleotides on the bottom of the tRNA, labeled as the anticodon, bind to the mRNA on the ribosome. The tRNA kit includes the ribosome mat for demonstrating translation as shown in Figure 6 and Figure 7. To assist in visualizing the steps before using the models, instructors showed a five-minute MIT Edgerton Center video where a pair of hands correctly models translation on the ribosome mat [26]. In Figure 6, the two green overlapping ovals represent the large and small ribosomal subunits, which unite to form the working ribosome in the cytoplasm. The ribosome mat includes several orange shapes, marking the position for the mRNA and tRNA molecules. Each tRNA molecule moves through three locations on the ribosome, known as the E, P, and A sites, labeled on the ribosome mat.

The messenger RNA molecule, responsible for selecting the order of the amino acids, binds to the ribosome first. Next, the tRNA molecules carrying specific amino acids bind to the mRNA (Figure 7). In Figure 7a, the arrow indicates that the protein chain comes off of the tRNA in the P site. That chain will bind to the end of the amino acid held in site A. In Figure 7b, a peptide bond is formed at the A site. This action shows how elongation of the protein chain occurs. The blue amino acid (Arg) stays attached to its tRNA in site A. In Figure 7c, both the mRNA and tRNA with the protein chain have moved over to the left by three nucleotide spaces (compared to Figure 7b). This move causes the empty tRNA to exit via the E site. This movement also opens up the A site for another tRNA to enter. The next tRNA selected will have an anticodon that base-pairs with AGC on the mRNA. This process continues until a release factor binds to the mRNA, releasing it from the ribosome.

### 2.5. Teacher Training Videos and Classroom Resources

The resources provided with MIT DNA and Protein Sets serve as excellent materials for self-instruction, strengthening faculty knowledge in preparation for teaching genomics. Additionally, training videos posted on the MIT Edgerton Center website demonstrate classroom instructional techniques for each set; also posted online are 12 three-minute videos for the hands-on activities. One example shows how to put together a protein chain from individual amino acids and how to fold the chain into an alpha helix. These practical and quick videos ensure that participants can build the structures, permitting them to focus on the concepts. The videos also show common building errors that help students avoid time-consuming missteps, assisting the instructor in keeping the class together. Summarizing videos are also provided and shown after the hands-on activities. Reviewing concepts with the videos strengthens the experience, helping the students recognize which facts are most important to learn. Lastly, students if absent can make up class time on their own if provided with a modeling kit, access to online videos, and the instructional booklets. All videos are listed on the MIT Edgerton Center DNA and Protein Set YouTube playlist [26].

### 2.6. A Case-Based Study with Cytochrome P450 (CYP450) Proteins

Cytochrome P450 (CYP) proteins, found in abundance in liver and kidney, help to break down toxicants and drugs by transforming them into water soluble molecules that can be excreted in the urine. However, CYP proteins can also convert relatively non-toxic molecules into potent DNA-damaging agents that cause cancer. A key concept for health professionals to recognize is that different world populations have variations (polymorphisms) in their CYP genes. These gene variants can have consequences in protein structure, making a population less or more sensitive to a specific toxicant, influencing health outcomes. A new case-based lesson using the MIT protein models was devised that presented three hypothetical examples (Carey, Yeshna, and Paxton) with CYP protein variants and where participants were guided to explore possible genetic susceptibilities. The protein variants illustrated in the examples were based on well-known CYP variants [27,28,29,30,31,32,33,34,35,36]. Native American populations in the U.S. were selected as relevant examples for the participants in the learning framework and for worldwide indigenous populations, more broadly.

**Figure 8:**
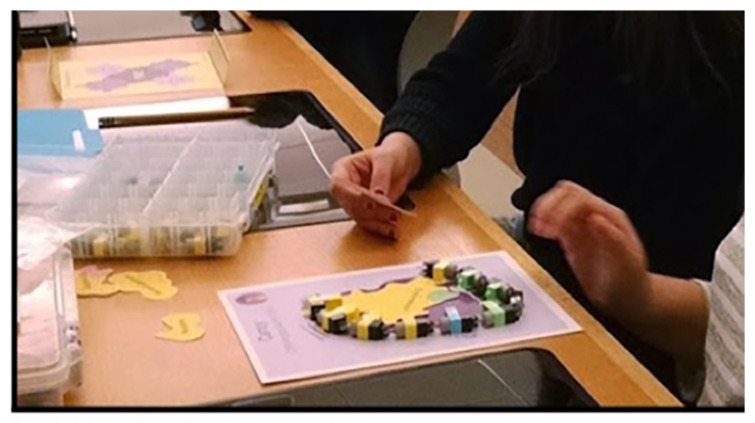
Participants are actively testing possible drug and toxicant binding sites inside a CYP protein. Drug and toxicant substrates are paper cards inserted into the CYP protein’s active site. Hydrophobic and hydrophilic properties, as well as the size of the substrate molecule, govern the CYP protein’s ability to bind the substrate and initiate the metabolic reaction.

After completing the introductory protein activities, nurse participants explored CYP protein function by constructing a two-dimensional representation of the protein’s active site. As shown in Figure 8, construction of the active site with the amino acids was guided by individual CYP Protein Mats, and participants worked in teams of two. As substrate binding initiates the metabolic process, each active site was tested for its ability to bind a specific environmental chemical or drug. The toxicant substrates were designed as color-coded paper cards to be positioned inside the active site. Hydrophobic and hydrophilic interactions, as well as substrate size, determined whether binding could occur. Polycyclic aromatic hydrocarbons (PAHs) were provided as a classroom example as this substrate can be converted into a potent carcinogen by the metabolic action of specific CYP proteins [33,34,35,36].

**Figure 9:**
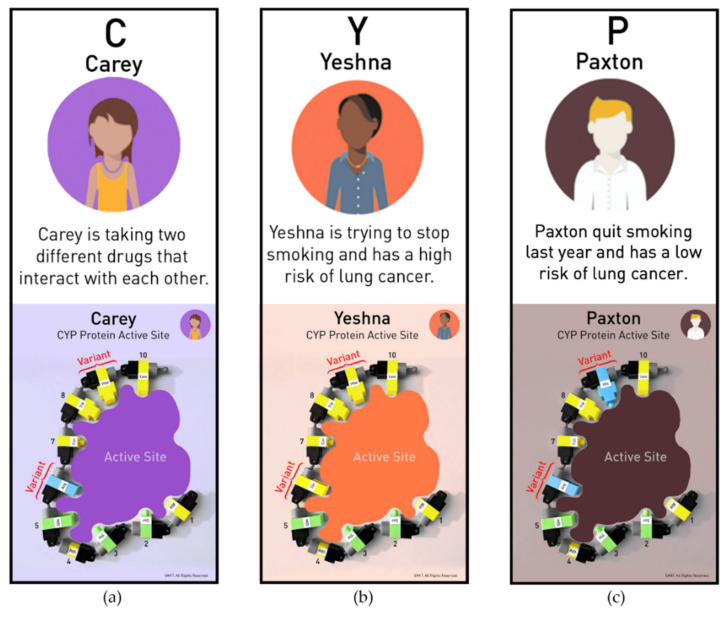
Carey (**a**), Yeshna (**b**), and Paxton (**c**) represent different world populations in a case-based scenario. Their stories before the activity are summarized here. The three CYP Protein Active Site Mats show the amino acid variants in their two-dimensional active sites. © MIT Edgerton Center.

Case studies with Paxton and Yeshna have two amino acids clearly marked as variants on their CYP Protein Mats (Figure 9). These variant amino acids and the ethnic heritage for each person match the research for their variant CYP proteins. Multiple CYP protein active sites were combined and simplified into two-dimensional structures to represent the CYP active site of each person. However, the models accurately depict the CYP proteins’ hydrophobic or hydrophilic interaction with the substrates. The models capture the altered interactions of the amino acids in the active site as well as amino acid sequence variations leading to the change in the proteins’ function.

Case-based studies for the study participants included representative scenarios to illustrate genetic susceptibility to environmental toxicants and drugs. Figure 9b,c, illustrates a case-based study that compares Yeshna’s (Figure 9b) and Paxton’s (Figure 9c) experiences with exposure to cigarette smoke. Because of their CYP variants, they will have different health outcomes.

**Figure 10:**
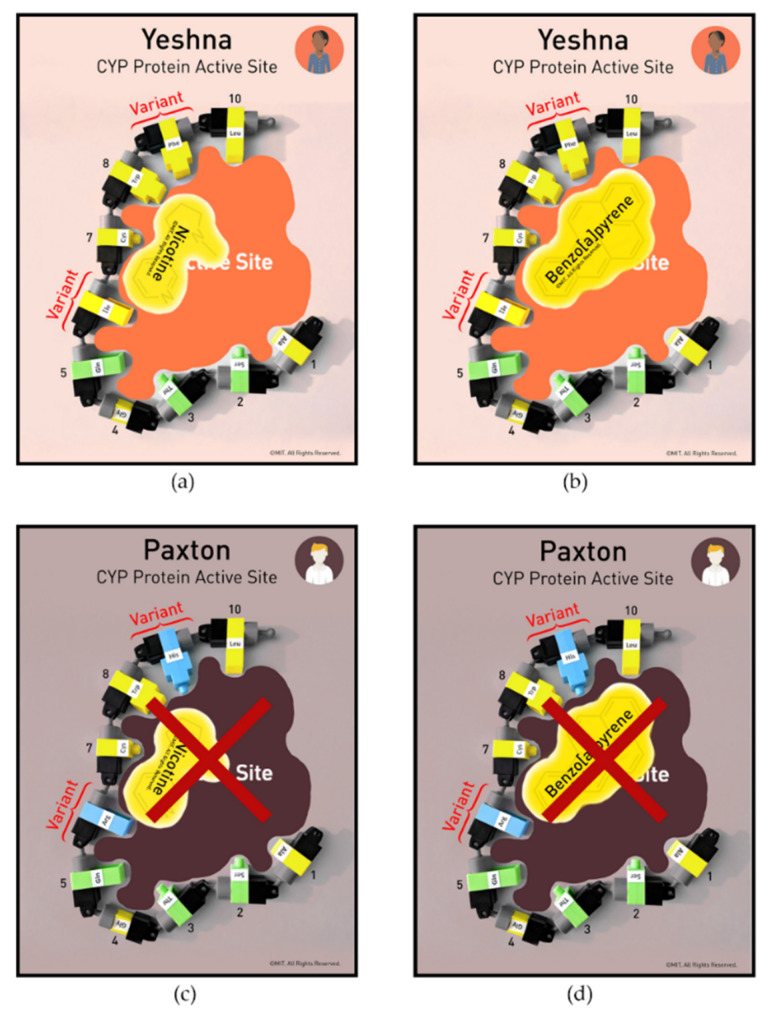
Nicotine and benzo[a]pyrene bind differently for Yeshna (**a**,**b**) than for Paxton (**c**,**d**). © MIT Edgerton Center.

The study participants discovered that Yeshna’s CYP protein binds and metabolizes nicotine easily and it leaves her body quickly. The broad hydrophobic patch of amino acids (in yellow) provides multiple sites for nicotine to bind (Figure 10a,b). Yeshna’s metabolism makes her have more frequent cravings for nicotine and she has greater difficulty quitting her smoking habit. Yeshna’s CYP protein active site also binds benzo[a]pyrene well as a result of its hydrophobic interactions with this substrate. Thus, a higher percentage of the benzo[a]pyrene molecules are converted into potent cancer-causing agents. This process explains why Yeshna has a higher risk of getting cancer from smoking.

In comparison, Paxton has an easier time quitting smoking and a lower risk of lung cancer (Figure 10c,d). The two hydrophilic amino acid variants (in blue) prevent the hydrophobic nicotine from binding well and it is metabolized poorly. Because nicotine stays in Paxton’s body longer, his nicotine cravings are decreased. In addition, his CYP protein binds and metabolizes benzo[a]pyrene poorly and produces fewer carcinogens.

These case studies, combined with tactile learning, lead to a conclusion that genetic variations in CYP proteins can affect human behavior, as well as cancer risk. After the students had worked with the models and discovered the potential health effects caused by genetic variations in CYP proteins, research results in figures graphically representing CYP2A6 variant distributions across different world geographic populations (e.g., Africa, Southeast Asia, and the U.S.) were presented for discussion [32]. The students noted the distribution of genetic variants in CYP protein types in these world populations were different. Genetic variations in populations produce changes in CYP protein amino acid sequences, causing major disparities in drug reactions, addictive behaviors, and cancer risk.

An example of the distribution of one CYP variant found in the U.S. was examined more closely. CYP2A6 is the name of this protein, and the symbol (*) after the name indicates a specific variant. CYP2A6 is the CYP protein that increases smoking desire by decreasing nicotine levels in the blood. More than 60% of the tested North Plains and Southwest Native American populations have the variant CYP2A6*1B compared to ~30% of Caucasian populations as in the case with Yeshna. In the case study presented to the students, Yeshna’s CYP proteins were modeled after typical Native American protein variants and Paxton’s CYP proteins were modeled after typical Caucasian protein variants [29,30,31,32].

### 2.7. Study Design and Content for the Models

To ensure familiarity with molecular representations and to teach the molecular content pertinent to genetics and environmental heath literacy, the hands-on component included the following four lessons: (1) protein structure and function, (2) DNA structure and function and mRNA transcription, (3) protein synthesis including translation on the ribosome, and (4) case studies on genetic susceptibility. The study session was three hours in duration, including the pre- and post-test times. Additionally, a ten-minute break was given halfway through the session.

### 2.8. Survey Method for the Models

The survey method for this study employed the MIT DNA and Protein Set with a pre-test–post-test method in which data were collected from participants at the beginning and end of the session. Data were collected from two tribally-affiliated sites. As one of the sites did not have classroom access to the internet for test taking online, data were collected uniformly at both sites by distributing printed questionnaires. This method of administrating the tests avoided any potential bias. To assure confidentiality, each participant was given instructions for creating a unique alphanumeric identifier code and asked to use the same code on both the pre-test and post-test versions. The questionnaire was primarily multiple choice with some true/false questions. In the pre-test, a brief set of open-ended questions were included concerning demographics and prior education in the topics to be taught.

Data were entered into Excel spreadsheets by the Tufts University CTSI staff. Responses were entered twice to check for accuracy. Using an answer key provided by the principal investigator from MIT, a scoring algorithm was created in which each correct answer was awarded a point. The 18-item questionnaire was scored from 0 (lowest score) to 17 (highest score). The “difference” was the post-test score minus the pre-test score.

### 2.9. Study Sites and Subjects

Two geographic sites were involved: Site 1 was the Ramapo College Nursing Program, in Ramapo, New Jersey, where advanced undergraduate nursing students in the Bachelor of Science in Nursing (BSN) Program support public health programs for the citizens of the Ramapo Lenape Tribe. Site 2 consisted of a diverse group of health professionals from the Cheyenne River Sioux Reservation. The primary partner at this site was the Missouri Breaks Industries Research, Inc. (MBIRI) in Eagle Butte, South Dakota, which is a research center employing 35 nurses. Our Project Team also partnered with the Oglala Lakota College, Eagle Butte Campus Associate Nursing Program where additional students and instructors participated. The pre-test and post-test data from the New Jersey and South Dakota sites were designated as “Nurse Data Ramapo (Site 1)” and “Missouri Breaks (MBIRI) (Site 2)” respectively, and presented together (*n* = 116).

### 2.10. Targeted Lectures in Toxicology

Our educational framework to improve public health knowledge includes targeted lectures on environmental health and toxicology in combination with the hands-on modeling exercises and case-based lessons. These lectures included the authors’ community-based environmental health research among Indigenous peoples of the U.S. At the Ramapo College in NJ, the nursing students received three environmental health/toxicology lectures (each ~40 min in duration with 20 min for questions and answers): (1) Environmental Health and Toxicology Basics, (2) Marginalized Communities and Environmental Contamination and Intervention Strategies, and (3) Metabolism of Environmental Toxicants. Similarly, three lectures were given at the Oglala Lakota College in South Dakota, on the Cheyenne River Sioux Tribal Reservation. Two presentations were also given to community members. One was an open evening lecture sponsored by Oglala Lakota College, and the second was for high school students given during the school day at Wakpala High School on the Standing Rock Tribal Reservation. An audience feedback form was piloted with the high school students to survey self-reported learning about health and the environment.

**Figure 11:**
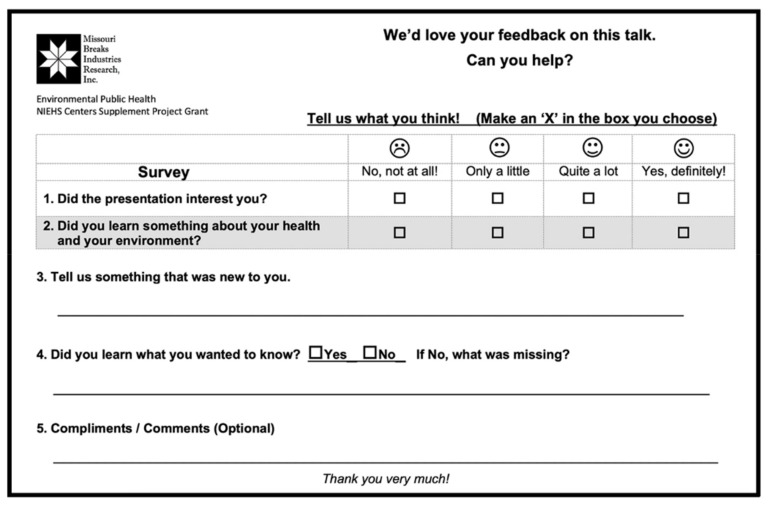
A modified SAMI Card for the community lecture survey. The format here is a simple paper-and-pencil task with four questions. An optional response line was included for a compliment or a comment.

The feedback form, on a half-page of paper, was a modified SAMI Card, an all-purpose assessment form originally created by the St. Louis Science Center (Figure 11). Our four-question, five-minute survey for this pilot was brief with a space for optional comments: two were Likert scale questions with four option-check boxes. Emoji faces clarified the ranking system [37,38]. The first Likert question measured interest in the lecture topic, and the second question measured self-reported learning about health and the environment. The next two questions were open-ended: “Tell us something that was new to you,” and, “Did you learn what you wanted to know?”

## 3. Results

### 3.1. Nurses’ Workshop with Hands-On Models: Participant Description

Results from the hands-on modeling workshops and environmental health lectures were collected from two study Sites: Site 1 from the New Jersey Ramapo College Nursing Program, Mahwah, NJ and Site 2 in Eagle Butte, SD. The data from the Ramapo Nursing Program together with the MBIRI site included a total of 116 participants. As shown in Table 1, the majority of participants in the study were young adult females and most self-identified as white. Among the learners, Asian/Pacific Islander and multi-racial groups were well represented. Participants’ prior educational experience in the field of genetics was also characterized.

### 3.2. Nurses’ Workshop with Hands-On Models: Statistical Analysis

The statistical analysis includes frequencies of each questionnaire variable in a combined analysis from both study sites. Frequencies are presented as numbers of participants and percentage (for categorical variables) and means and standard deviations (for continuous variables). Differences between study sites were not assessed. Pre-test and post-test change scores at the questionnaire item level and total score were tested with *t*-tests. A statistically significant change is *p* < 0.05.

Results of pre-test to post-test change in the scores from both sites are shown in Table 2. There was a statistically significant improvement in the score from a pre-test mean of 10.9 (SD = 2.2) to 14.0 (SD = 1.8) in the post-test mean score with the mean difference of 3.1 (SD = 2.0; *p* < 0.0001). The full 18-item questionnaire including a description of the developmental process can be found in the Supplementary Materials.

Table 3 presents the results from four sample test questions from pre- and post-tests, which demonstrate the greatest improvement in environmental health-related knowledge. Improvements between the pre-test and post-tests values are shown for specific test questions with the correct answer indicated in green.

### 3.3. Lecture Survey: Community Youth Participants and Results

To obtain feedback on a public health lecture presented to Tribal community members, a SAMI Card survey form (Figure 11) was administered at the Wakpala School in South Dakota. Fifty high school students of all genders, grades 9–12, from the Standing Rock Reservation gathered to hear a 20 min lecture on the science of vaping as requested by the school administrators. The lecture was presented in the school auditorium by the scientist from New York University Langone School of Medicine. Because our MBIRI partner in South Dakota is a local research organization managed by a Tribal nation, our team was invited to present community health lectures at several other venues in the surrounding Tribal reservations. We chose the Wakpala School to launch our lecture survey.

Using the SAMI Card as a survey tool, the results summarized in Table 4 showed that interest in vaping was very high among the youth participants (92%). Likewise, when the students were asked about whether they “had learned something about health and [their] environment,” their responses were also highly positive (98%). When asked about something new they had learned, the majority (66.7%) described how vaping affects human health and/or why vaping is bad for you, while others described vaping products as new information. For the response on whether they “had learned what they wanted to know about vaping,” 96% agreed that they had. Overall, the participants were receptive about learning how their behaviors and environment affect their health.

## 4. Discussion

### 4.1. Previous Studies

The nursing students’ robust educational gains reported here further substantiate an earlier study published in the *Journal of Nursing Education* by the authors and their extended team [39]. This earlier study achieved learning gains with the same pre- and post-tests significance values of *p* < 0.0001, demonstrating the hands-on learning approach is well-suited to teaching the molecular basis of genetic susceptibility. This earlier study also demonstrated the modeling was effective across a broad nursing audience, as nurses were of different ages, educational and cultural backgrounds, and were employed in numerous nursing fields. These earlier workshops, held annually on the MIT campus between the years 2007 and 2012, attracted practicing nurses from major hospitals, research institutions, and also faculty from nursing schools across the U.S. The workshops’ purpose was to teach key molecular concepts in genetics/genomics and to offer participants Continuing Education Units (CEUs) for the workshop; U.S. nurses are required to earn CEUs annually by state rules. These studies served as the basis for an expanded environmental health educational framework presented here [39].

The previous nurse workshops conducted on the MIT campus also explored the long-term recall of the molecular biology concepts, asking participants for a personal or work-related example of how they had used their newly acquired genetic knowledge. An email request was sent to 50 participants who had attended workshops one or two years prior. In this way, the two cohort groups completing responses were replying three to fifteen months after their workshops. The survey returned 12 volunteered online responses, including the three examples quoted here: (1) “Articles that I read make so much more sense to me now,” (2) “In reviewing new research protocols, I have a better understanding of the mechanism by which the drug’s action is being researched”, and (3) “I have been able to explain the changes occurring in the flu viruses to people who do not understand the importance of vaccination” [39]. In the latter example, the practicing nurse reported she could now, knowing more about mutations and genetics, explain the need for annual flu vaccinations.

Taken together, the results of previous and current studies support the anticipated outcome that hands-on experiences with the MIT DNA and protein models lead to an increase in nurses’ scientific literacy. From previous studies it has been shown that nurses retained these knowledge gains through specific situations where they applied genetic concepts. Examples given included continuing educational endeavors such as reading research journals, and communicating difficult-to-understand public health information to patients, such as explaining why flu vaccinations are needed on an annual basis.

### 4.2. Major Findings and Implications from Current Work

Nursing students in the current study experienced the same hands-on genetics lessons as in the prior study, but in addition, we piloted the case-based CYP protein lesson demonstrating genetic susceptibility, as previously detailed in Methods and Results. The authors plan to continue developing curricula utilizing the modeling kits to explain additional genetics and environmental health concepts. In this way, the next generation of nurses will be better prepared to care for marginalized populations who live on lands where environmental justice contamination issues are present. Additionally, because nurses learn in our framework by actively practicing with genetic models, this educational approach is well positioned for international adoption. “Learning by doing” is a universal teaching method and it relies less on language for conveyance of essential concepts [19,20,21,22].

In both MIT studies, instructors taught protein synthesis in a novel and non-traditional manner. Protein synthesis, also known as the “central dogma of molecular biology” is summarized as: “DNA ➞ RNA ➞ protein.” Thus, biology teachers have traditionally organized their curriculum in this order. They begin by teaching DNA, continue by describing the role of RNA, and complete the lesson with the creation of a protein. However, since students are unfamiliar with protein structure and function, learning this multistep process is confusing. In contrast, the MIT curriculum introduces proteins first, before DNA. This pedagogical innovation results in improving students’ grasp of the process. In our curriculum, students first build, fold, and place a model channel protein into a mock cell membrane. Then students simulate protein synthesis (DNA ➞ RNA ➞ protein), constructing the same channel protein again. By teaching the structure and function of this channel protein first, students understand the process of protein synthesis more easily and can anticipate the amino acid sequence required for a functional channel protein as well.

Proteins are consistently neglected in secondary education. Evaluations and testimony from high school biology teachers teaching protein synthesis using our models and the “proteins first” method report that it took less time to teach protein synthesis, and that their students grasped the concepts better [40]. One suggestion offered by Vandiver as to why proteins remain unfamiliar to students is that educators have been hampered by lack of tactile protein models. Contrast this absence with the abundance of DNA models commercially available from educational supply houses. Additionally, DNA is visually present in multimedia, and its iconic structure is well recognized.

### 4.3. Discussion of Nurses’ Learning Gains

Results from the current study revealed nursing students’ weak foundational knowledge about proteins and DNA. Approximately one quarter of the students did not initially recognize proteins as essential cellular components. Constructing two model proteins with important cellular roles produced a significant increase in students’ understanding. The pre-tests also revealed nursing students’ initial confusion with DNA organization, including the terms: genes, chromosomes, and nucleotides. Excellent learning gains were recorded for these key definitions by visualizing the physical nucleotide models and using them to create genes.

A lack of knowledge about the role of proteins in health was also evident, as recognition of cytochrome P450 enzymes’ function in metabolic homeostasis was initially low. In addition, the importance of genetic variation in protein function was not universally recognized. Overall, the post-test responses indicated that the case-based lesson using the protein models effectively demonstrated both CYP protein function and the effects of genetic variations on health outcomes. A deeper understanding of CYP proteins is particularly useful to healthcare professionals, assisting them in recognizing why some people are more sensitive to chemical exposures than others.

A revolutionary advantage of working with the models could be dismantling one of the psychological barriers to biochemistry. Proteins are typically introduced to students as atomic chemical structures. Chemistry’s symbolic language can be both intimidating and difficult to visualize. The models are more cognitively inviting with their colors and shapes that evoke a sense of playfulness, reducing a fear of failure. Overall, the learning process is far more engaging, and the opportunity for hands-on practice provides for greater feedback, which helps students to assemble their own cognitive images for a deeper understanding. Additionally, students working with the kits in pairs can assist and support each other. Anecdotally, instructors noted that even the less interested students asked questions about the biological processes.

### 4.4. Community Education Outreach and Applicability

Although the major focus of this project was teaching healthcare professionals serving Indigenous populations, in response to requests of our Tribal partner at MBIRI (Eagle Butte, SD, USA) the researchers piloted additional methods to reach Tribal youth and community members. The authors presented lectures and provided hands-on lessons at four different school systems in the region, including Timberlake HS (Timberlake, SD, USA), Windswept School (Eagle Butte, SD, USA), Wakpala School (Mission Township, SD, USA), and Dupree HS (Dupree, SD, USA). Additionally, the Oglala Lakota College in Eagle Butte hosted a presentation provided by one of the authors, speaking on environmental health and toxicology, which drew a large number of community members as well as college students to an evening event. These sessions were encouraging, and the authors plan to do more community-based events with Tribal partners and rural populations in the future.

## 5. Conclusions

Data gathered through pre- and post-tests returned statistically significant improvement in knowledge gains following hands-on instruction with the MIT DNA and Protein Sets. Learning occurred in diverse settings as shown among 116 nurses serving Native Americans living both in urban and rural areas of the U.S. These results corroborated our prior study results [39]. Furthermore, the case-based modeling lessons deepened the nursing students’ understanding about genetic variants in CYP 450 proteins common in various ethnic populations and their corresponding health vulnerabilities. Our major achievement was demonstrating the utility of our educational framework across diverse populations and remote communities. The data showed that the modeling lessons clearly increased nursing students’ awareness of the effects of environmental toxicants, a key step toward improving health care for exposed communities. This work also included high school students and their teachers, and our program can easily be extended towards higher nursing and elective medical school programs. Our educational tools with their environmental health focus make this educational framework applicable for worldwide dissemination, and especially relevant to healthcare organizations working with populations subjected to long-term exposure to environmental hazards.

## 6. Patents

1. Casarez B.L., Vandiver K.M., & Vandiver J.K. (2014). *Educational Building Blocks to Model DNA and RNA Structures.* (Patent US20160133157 A1). U.S. Patent and Trademark Office.
2. Vandiver J.K. & Vandiver K.M. (2019). *Educational Building Blocks to Model Protein Assembly from Amino Acids.* (Patent US10410540 B2). U.S. Patent and Trademark Office.

## Figures and Tables

**Table 1:** Demographics of participants from both study sites. Table includes gender, age, ethnicity, and prior biology coursework. Results include the number of participants followed by the percentage of the total participants in parenthesis.

Participant Characteristic	Participants Grouped by This Characteristic (*n* = 116)
Female	93 (80.2%)
Mean Age (SD)	22.5 (6.9)
Hispanic	12 (10.4%)
**Race/Ethnicity:**	
Asian/Pacific Islander	19 (16.5%)
Alaskan	0 (0%)
Black	4 (3.5%)
Native American	4 (3.5%)
White	76 (66.1%)
Other/Multi-racial	12 (10.4%)
Missing	1
**Undergraduate Courses:**	
Introductory Biology	13 (11.2%)
Several Science classes	87 (75.0%)
Genetics + Other Science	12 (10.3%)
Genetics + Job Experience	4 (3.5%)

**Table 2:** Study results of pre-test and post-test mean scores. The table includes the descriptive statistics.

Variable	N	Mean	Standard Deviation	Minimum	Maximum
Pre-test	116	10.9	2.2	5.0	17.0
Post-test	116	14.0	1.8	8.0	17.0
Difference	116	3.1	2.0	−6.0	7.0

**Table 3:** Selected pre- and post-test study questions and answers. Questions are in bold and correct answers are in bold italic. For each answer the number of responses is shown, followed by the percentage of the total of responses.

Selected Questions and Answers	Pre-Test Response N = 116 n (Percentages in Brackets)	Post-Test Response N = 116 n (Percentages in Brackets)
**Test Question 2: In the body, proteins are found:**		
Only in the brain	0 (0%)	0 (0%)
In extracellular fluid-like blood serum	9 (7.9%)	4 (3.5%)
***In all cells***	***88 (77.2%)***	***104 (91.2%)***
In some but not all cells	17 (14.9%)	6 (5.3%)
Missing	2	2
**Test Question 5: Genes are made of subunits called:**		
Chromosomes	70 (60.3%)	24 (20.9%)
***Nucleotides***	***41 (35.3%)***	***82 (71.3%)***
Amino acids	5 (4.3%)	9 (7.8%)
Missing	0	1
**Test Question 12: The liver degrades foreign molecules and drugs for elimination in urine. Liver cells accomplish this metabolic task with proteins called:**		
***Cytochrome P450 enzymes***	***10 (8.6%)***	***95 (82.6%)***
Serine P20 proteinases	5 (4.3%)	1 (0.9%)
Hepatic acetylcholinesterases	44 (37.9%)	9 (7.8%)
Hepatic hydrolases	57 (49.2%)	10 (8.7%)
Missing	0	1
**Test Question 17: True or False. Genetics heritage can determine a CYP protein’s ability to detoxify environmental poisons.**		
***True***	***97 (84.4%)***	***114 (99.1%)***
False	18 (15.6%)	1 (0.9%)
Missing	1	1

**Table 4:** Lecture results survey form. The survey question is highlighted in gray. For each question, the number of youth responses is shown, followed by the percentage of the total responses. For the write-in responses in Question 3, comments were grouped by topic and the percentage of the total comments for each topic were listed.

Survey Questions and Answers	Response N = 51 n (Percentages in Brackets)
**Likert Question 1: Did the presentation interest you?**	
Quite a lot	22 (43.1%)
Yes, definitely	13 (25.5%)
Only a little	12 (23.5%)
No, not at all	4 (7.8%)
Missing	0
**Likert Question 2: Did you learn something about your health and your environment?**	
Yes, definitely	21 (42.9%)
Quite a lot	18 (36.7%)
Only a little	9 (18.3%)
No, not at all	1 (2.0%)
Missing	2
**Open-Ended Question 3: Tell us something that was new to you.**	
Comments about what vaping does	18 (46.2%)
Comments that vaping is bad	8 (20.5%)
Comments about e-cigarettes vs. cigarettes	4 (10.2%)
Comments about vaping flavors	4 (10.2%)
Comments about different types of vapes	3 (7.7%)
Comments about e-cigarettes having nicotine	2 (5.2%)
Missing	12
**Question 4: Did you learn what you wanted to know?**	
Yes	36 (72.0%)
No	12 (24.0%)
N/A	2 (4.0%)
Missing	1
**Optional Youth Comments/Compliments (*n* = 11)**	
“Nice talk straight to the point”	
“Thanks for visiting our school”	
“Great job”	
“Pretty good”	
“Thank you for coming!”	
“I learnt a lot more about e-cigarettes”	
“I like your [New York] accent”	
“This was quite helpful to me”	
“You guys know a lot about e-cig”	
“Very informative”	
“I liked the presentation because now I know not to smoke or use vape.”	

## Data Availability

No additional data.

## Supplementary Materials

The following are available online at https://www.mdpi.com/article/10.3390/ijerph19020929/s1, Supplementary File S1. Reference [39] is cited in the Supplementary Materials.
