# Supplementary material for: Building Environmental Health and Genomics Literacy among Healthcare Providers Serving Vulnerable Communities: An Innovative Educational Framework
Source: Int J Environ Res Public Health. 2022 Jan 14;19(2):929. doi: 10.3390/ijerph19020929 (PMC8776035; doi:10.3390/ijerph19020929)
Supplement: Supplementary file 1 [file ijerph-19-00929-s001.zip › ijerph-1477916-supplementary.pdf]

Supplementary Materials

Questionnaire Development

The questions used in the current study (see Table S1 below) were first created for the two-day Nurse Workshops on Genetics and Environmental Health at MIT. The workshops were held annually from 2009 – 2014. The Nurse and MD participants were awarded 16 Continuing Education Units (CEUs) through the Harvard Catalyst Clinical and Translational Science Award (CTSA) at Harvard Medical School. The results from the workshops were published in the Journal of Nursing Education [39]. For the current study, several new CYP proteins and genetic susceptibility questions were added to this well-validated and successful questionnaire.

Table S1: Additional Pre- and Post-test Study Questions and Answers. Questions are highlighted in grey and correct answers highlighted in green. For each answer the number of responses is shown, followed by the percentage of the total of responses

**Additional Questions and Answers**	**Pre-test response N=116 n (percentages in brackets)**	**Pre-test response N=116 n (percentages in brackets)**
**Test Question 1: Proteins are made of subunits called:**		
Nucleotides	5 (4.4%)	3 (2.6%)
Lipids	0 (0%)	0 (0%)
Amino Acids[1]	110 (95.6%)	112 (97.4%)
Monolayers	0 (0%)	0 (0%)
Missing	1	1
**Test Question 3: A hydrophobic protein chain will tightly fold up when surrounded by:**		
Water	103 (88.8%)	111 (96.5%)
The nucleus	2 (1.7%)	1 (0.9%)
Ribosomes	4 (3.5%)	1 (0.9%)
Lipids	7 (6.0%)	2 (1.7%)
Missing	0	1
**Test Question 4: Proteins have many functions, but they do not:**		
Work as enzymes	11 (9.6%)	22 (19.1%)
Carry oxygen molecules	55 (47.8%)	46 (40.0%)
Make pores in cell membranes	27 (23.5%)	15 (13.0%)
Carry instructions for making new cells	22 (19.1%)	32 (27.8%)
Missing	1	1
**Test Question 6: Which base in DNA will correctly pair with thymine?**		
Adenine	97 (83.6%)	112 (97.4%)
Uracil	6 (5.2%)	0 (0%)
Cytosine	7 (6.0%)	1 (0.9%)
Guanine	6 (5.2%)	2 (1.7%)
Missing	0	1
**Test Question 7: DNA contains the codes for:**		
Fats	0 (0%)	1 (0.9%)
Proteins	113 (97.4%)	114 (99.1%)
Carbohydrates	3 (2.6%)	0 (0%)
Missing	0	1

Test Question 8: The genetic code utilizes three bases to code for one:		
Phospholipid	2 (1.7%)	0 (0%)
Triglyceride	24 (20.7%)	11 (9.6%)
Nucleotide	41 (35.3%)	39 (33.9%)
Amino acid	49 (42.3%)	65 (56.5%)
Missing	0	1
**Test Question 9: DNA molecules have two strands that can separate. The weak bond connecting these strands is called a:**		
Polar bond	17 (14.7%)	5 (4.35%)
Hydrogen bond	78 (67.2%)	106 (92.2%)
Covalent bond	21 (18.1%)	4 (3.5%)
Missing	0	1
**Test Question 10: Look at the DNA diagram below. Which side of the DNA is used to create mRNA?**		
The side that is the gene	48 (41.4%)	20 (17.7%)
The side that is opposite the gene	68 (58.6%)	93 (82.3%)
Missing	0	3
**Test Question 11: Creating mRNA from DNA is called:**		
Replication	15 (12.9%)	4 (3.5%)
Translation	31 (26.7%)	7 (6.1%)
Transcription	68 (58.6%)	104 (90.4%)
RNA splicing	2 (1.7%)	0 (0%)
Missing	0	1
**Test Question 13: Look at the diagram of mRNA attached to the ribosome. Which of the following is TRUE?**		
"Lys" will be added onto the "Met"	42 (36.2%)	7 (6.1%)
"Met + Pro" will be added onto the "Lys"	74 (63.8%)	108 (93.9%)
Missing	0	1
**Test Question 14: Look at the diagram of two protein chains below. Which statement is TRUE?**		
Chains are the same protein	11 (9.5%)	3 (2.6%)
Chains are two different proteins	105 (90.5%)	112 (97.4%)
Missing	0	1
**Test Question 15: Here are three statements about DNA Repair. Which statement is FALSE?**		
Cell proteins perform DNA repair	22 (19.0%)	7 (6.1%)
Oxygen can damage DNA	21 (18.1%)	21 (18.3%)
DNA never needs repair	73 (62.9%)	87 (75.6%)
Missing	0	1
**Test Question 16: Which statement about Mendelian genetics is FALSE?**		
A "recessive" trait can be caused by changes in protein function	44 (38.3%)	44 (38.3%)
A "carrier" is an individual who carries two normal genes for a trait	71 (61.7%)	71 (61.7%)
Missing	1	1
